# Supplementary figures and images for: Inhibition of xpt Guanine Riboswitch by a synthetic nucleoside analog
Source: PLoS One. 2025 May 5;20(5):e0322308. doi: 10.1371/journal.pone.0322308 (PMC12052177; doi:10.1371/journal.pone.0322308)

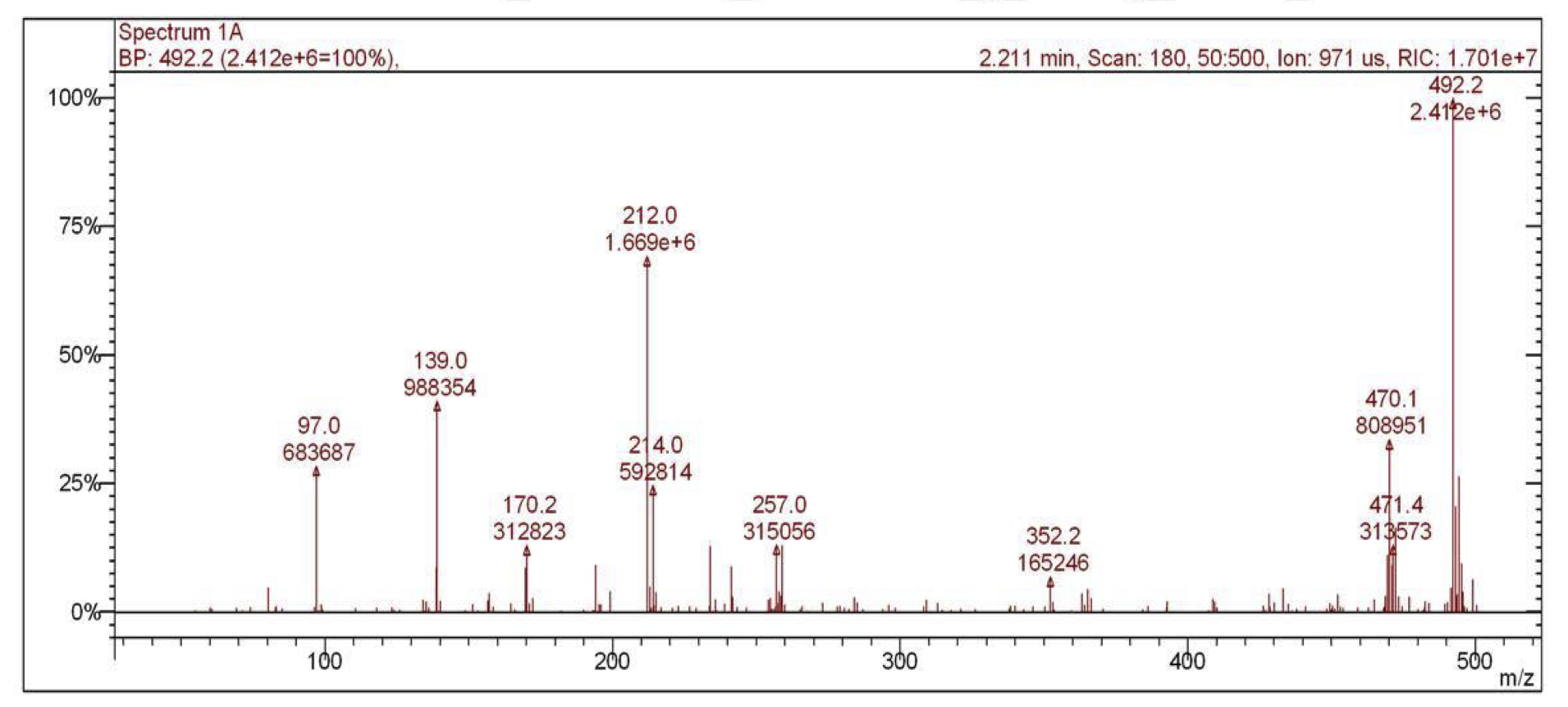

Supplement: S1 Fig — (TIF) [file pone.0322308.s001.tif]

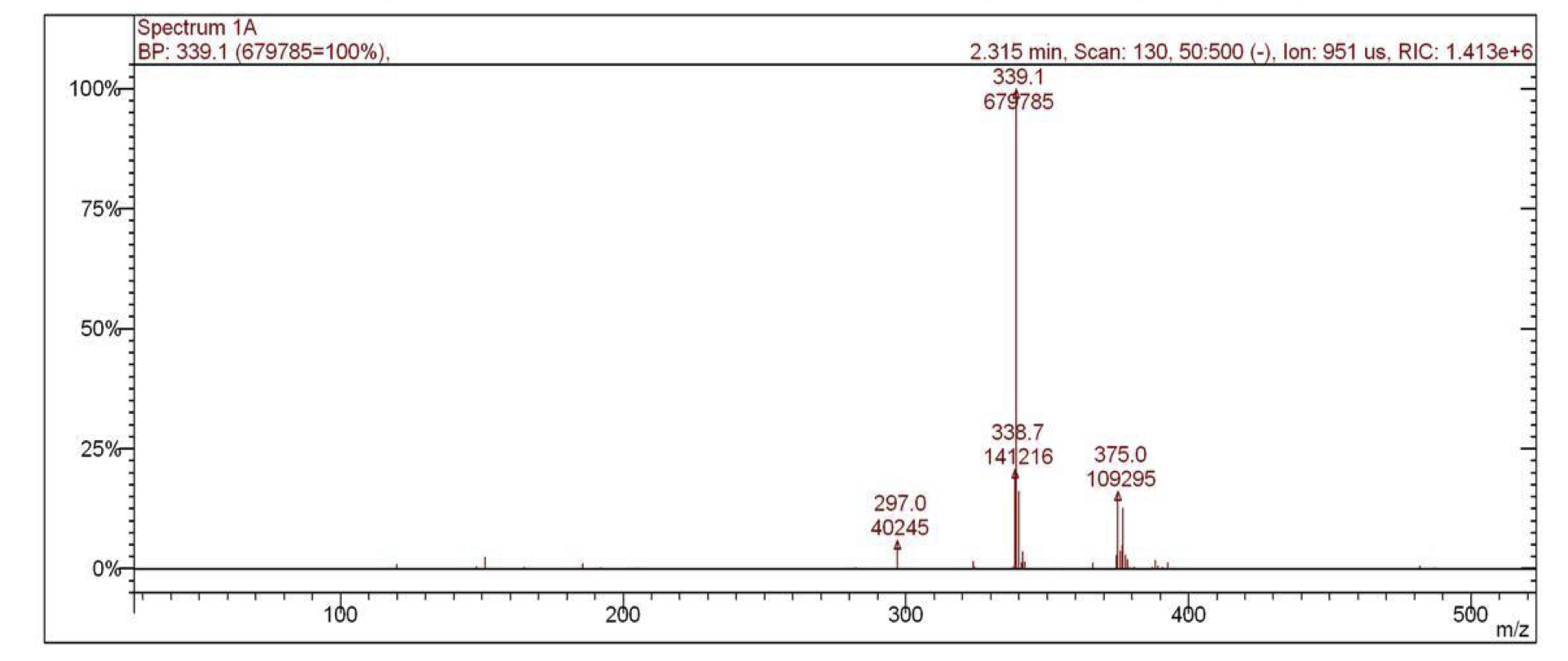

Supplement: S2 Fig — (TIF) [file pone.0322308.s002.tif]

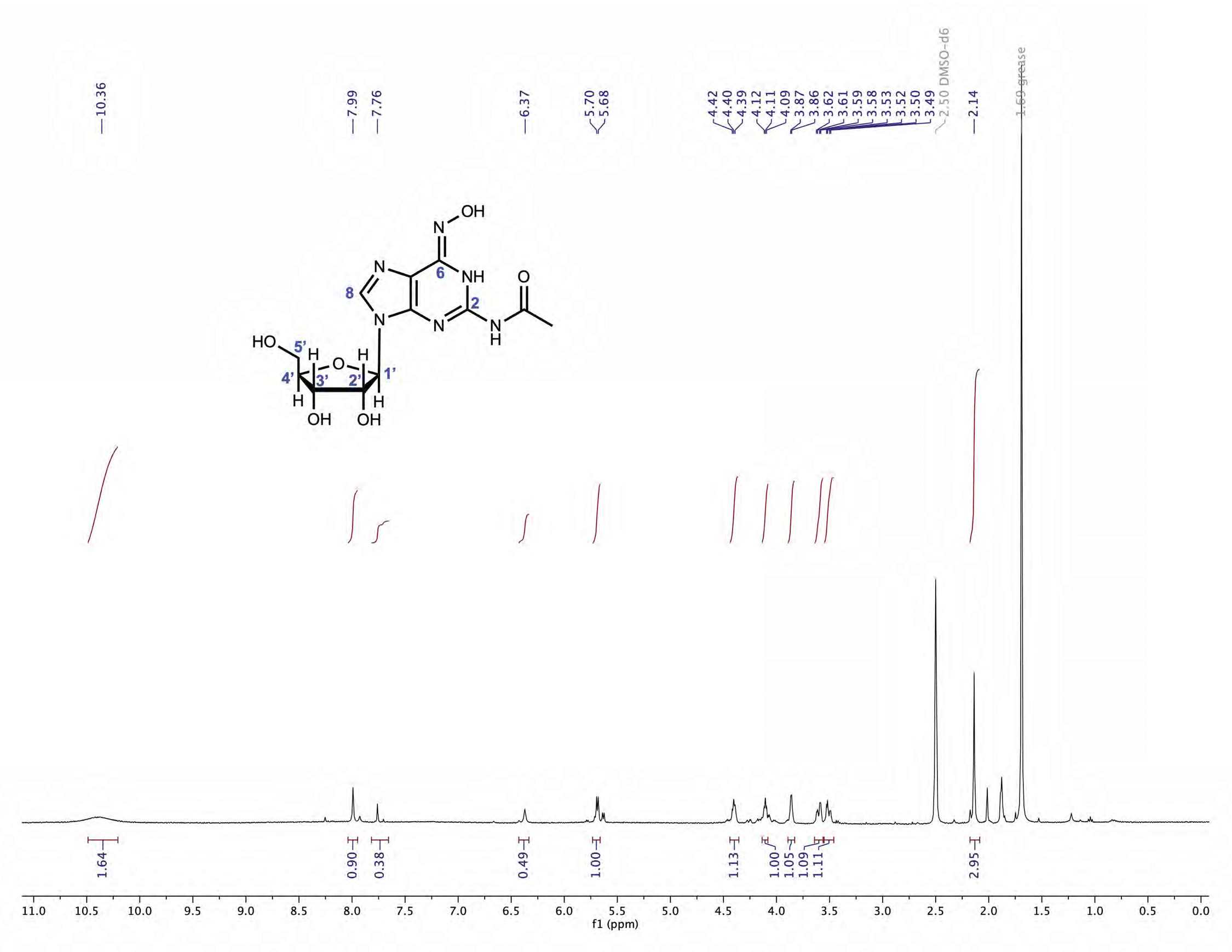

Supplement: S3 Fig — (TIF) [file pone.0322308.s003.tif]

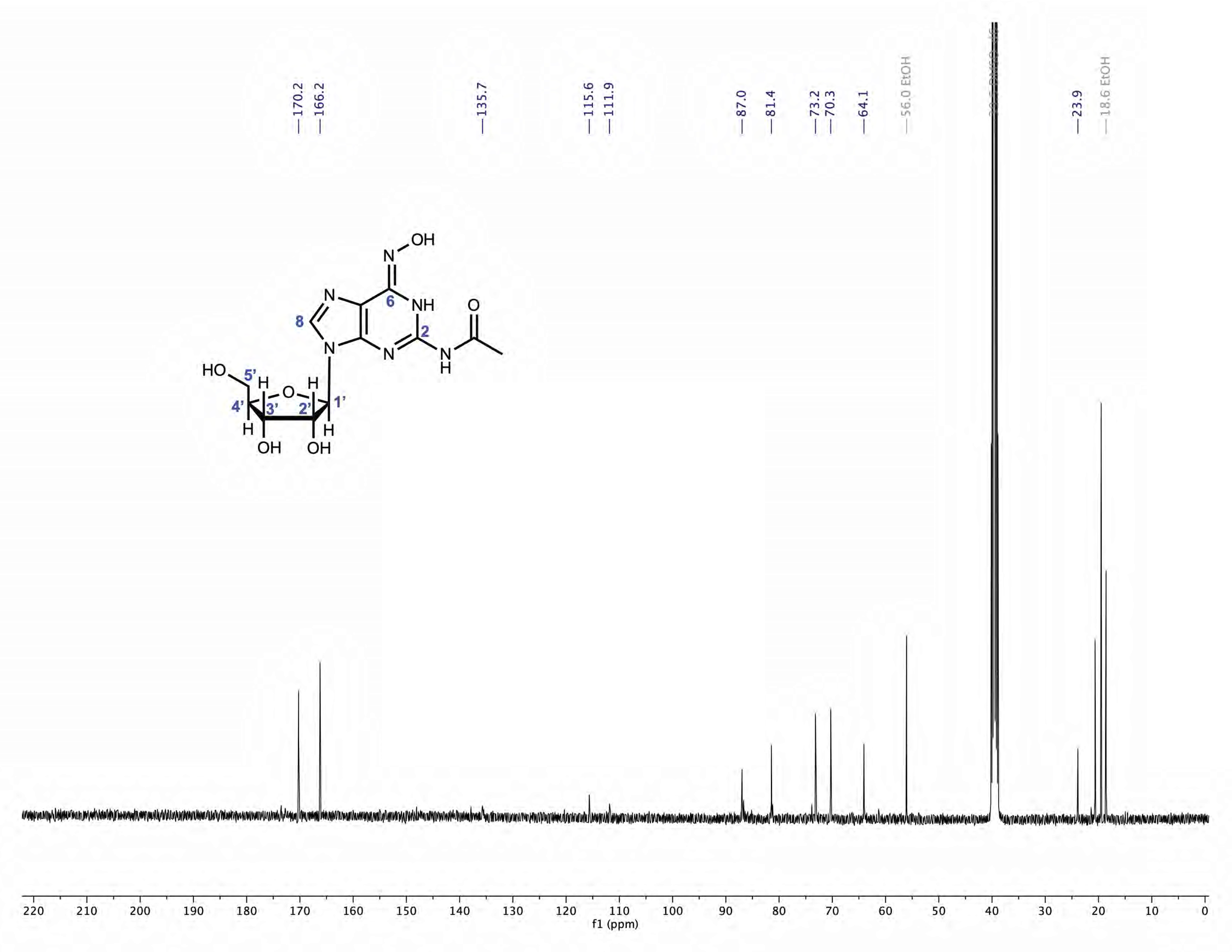

Supplement: S4 Fig — (TIF) [file pone.0322308.s004.tif]

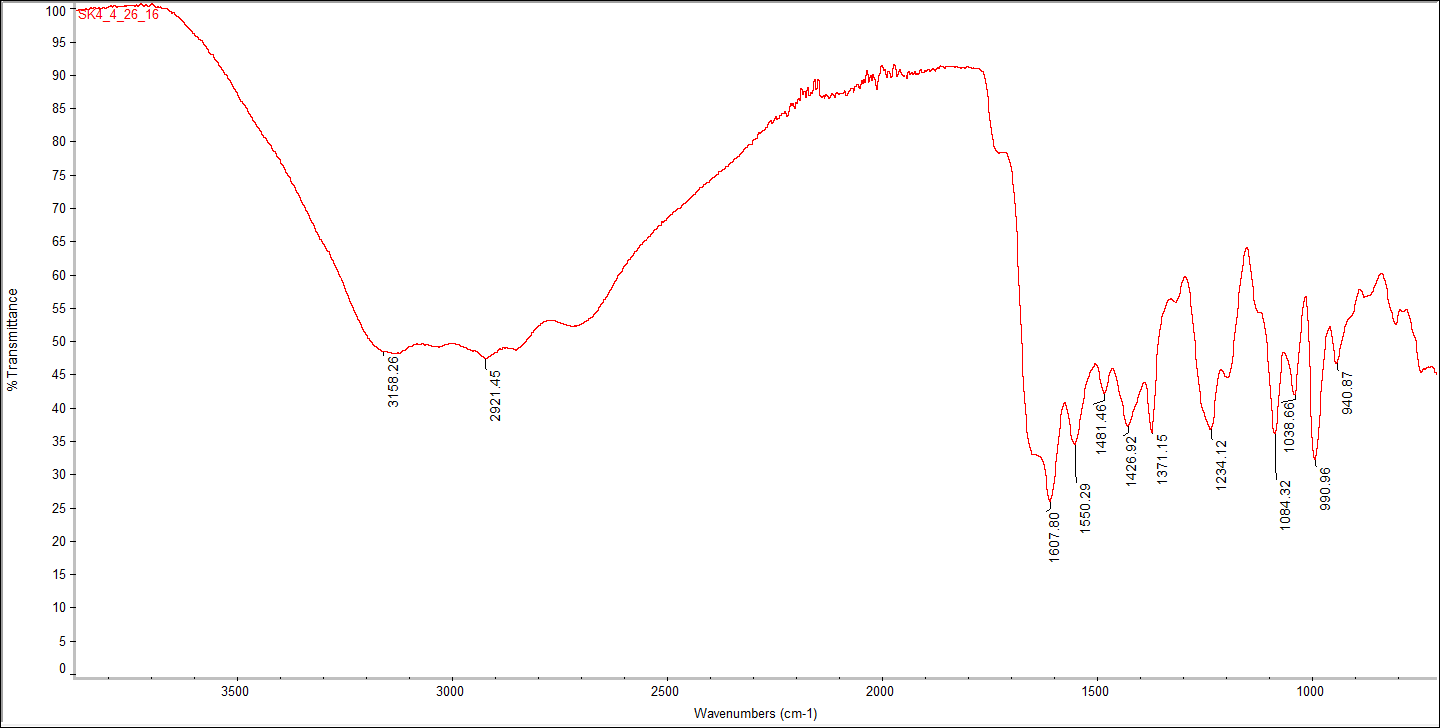

Supplement: S5 Fig — (TIF) [file pone.0322308.s005.tif]

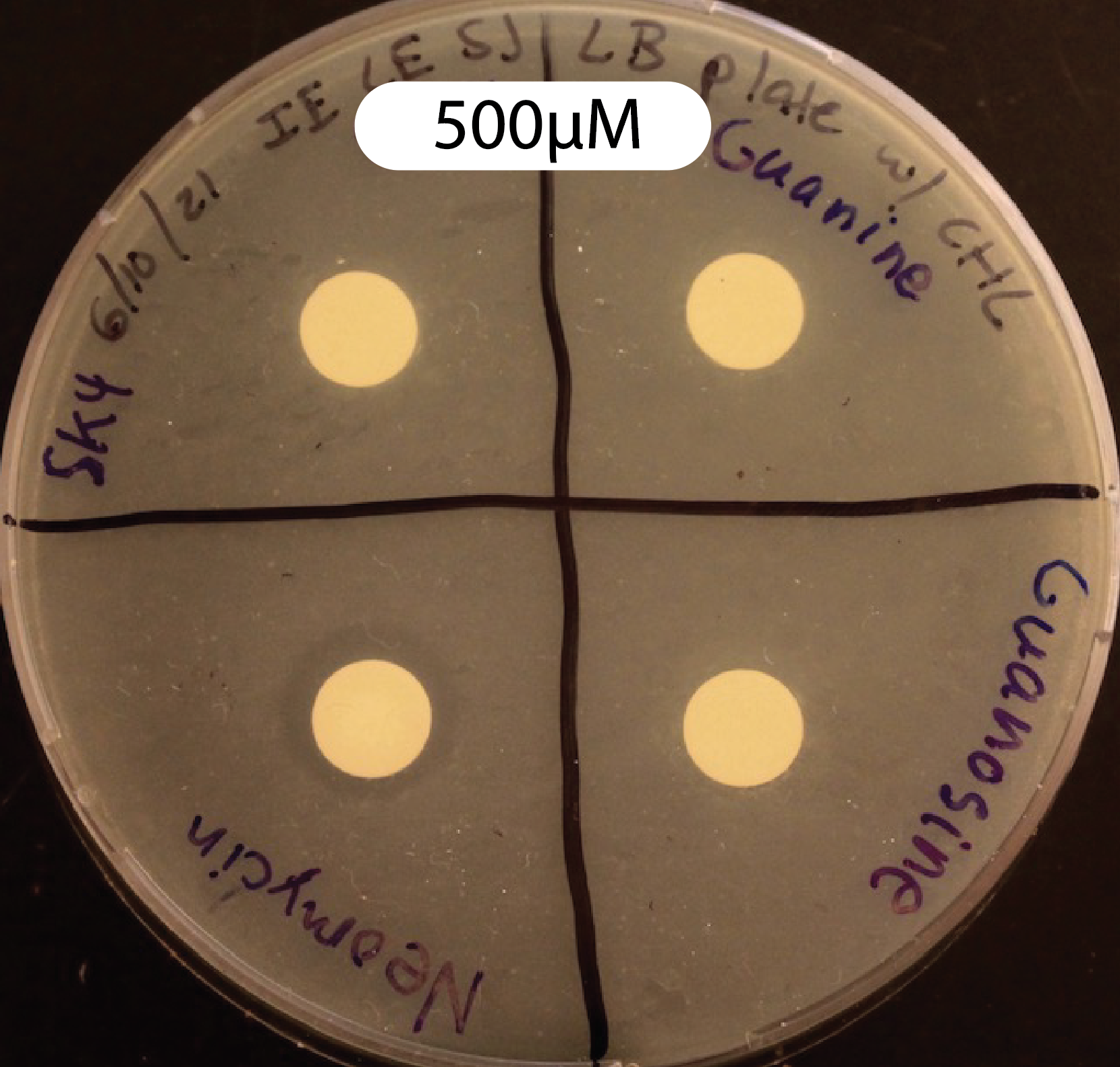

Supplement: S6 Fig — This figure shows that B. subtilis cells are viable at 500 micromolar guanine, guanosine, and SK4. Neomycin (with a zone of inhibition) is shown as a positive control. (TIF) [file pone.0322308.s006.tif]

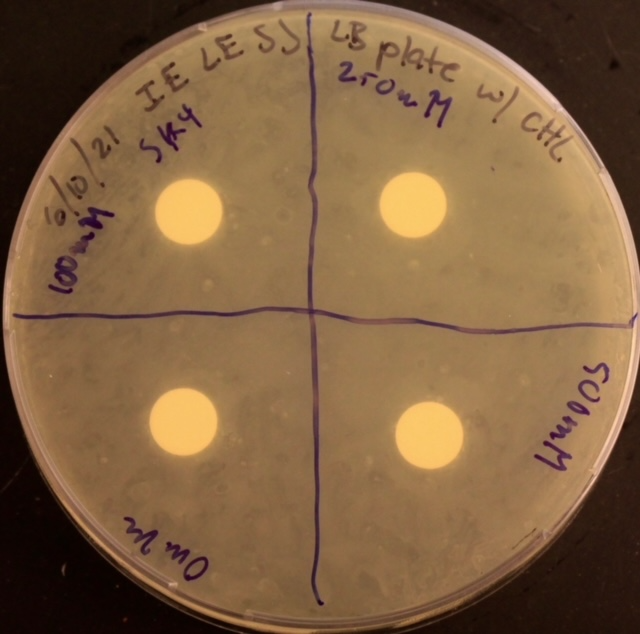

Supplement: S7 Fig — This figure shows that B. subtilis cells are viable (no zones of inhibition are observed) upto a concentration of 500 micromolar SK4. (TIF) [file pone.0322308.s007.tif]
